# Infection prevention and control in Dutch general practices before and during the COVID-19 pandemic and its implications for pandemic preparedness and seasonal respiratory epidemics: a qualitative study on lessons learned

**DOI:** 10.1186/s12875-024-02451-z

**Published:** 2024-06-20

**Authors:** Famke Houben, Casper D. J. den Heijer, Nicole H. T. M. Dukers-Muijrers, Eefje G. P. M. de Bont, Hanneke T. Volbeda, Christian J. P. A. Hoebe

**Affiliations:** 1 Department of Social Medicine, Care and Public Health Research Institute (CAPHRI), Faculty of Health, Medicine and Life Sciences, Maastricht University, P.O. Box 616, Maastricht, 6200 MD The Netherlands; 2 Department of Sexual Health, Infectious Diseases and Environmental Health, Living Lab Public Health MOSA, South Limburg Public Health Service, P.O. Box 33, Heerlen, 6400 AA The Netherlands; 3 Department of Medical Microbiology, Infectious Diseases and Infection Prevention, Care and Public Health Research Institute (CAPHRI), Faculty of Health, Medicine and Life Sciences, Maastricht University Medical Centre (MUMC+), P.O. Box 5800, Maastricht, 6202 AZ The Netherlands; 4 Department of Health Promotion, Care and Public Health Research Institute (CAPHRI), Faculty of Health, Medicine and Life Sciences, Maastricht University, P.O. Box 616, Maastricht, 6200 MD The Netherlands; 5 Department of Family Medicine, Care and Public Health Research Institute (CAPHRI), Faculty of Health, Medicine and Life Sciences, Maastricht University, P.O. Box 616, Maastricht, 6200 MD The Netherlands

**Keywords:** COVID-19, Infection control, Primary health care, General practice, Family medicine, Qualitative research

## Abstract

**Background:**

The COVID-19 pandemic has prompted a re-evaluation of infection prevention and control (IPC) in general practices, highlighting the need for comprehensive IPC implementation. This study aimed to evaluate healthcare workers’ (HCWs) experiences and perspectives regarding IPC in general practices before and during the COVID-19 pandemic, and its implications for post-pandemic IPC implementation.

**Methods:**

This qualitative study involved semi-structured, in-depth interviews during two time periods: (1) prior to the COVID-19 pandemic (July 2019-February 2020), involving 14 general practitioners (GPs) and medical assistants; and (2) during the COVID-19 pandemic (July 2022-February 2023), including 22 GPs and medical assistants. Data analysis included thematic analysis that addressed multiple system levels.

**Results:**

Findings indicated a shift towards comprehensive IPC implementation and organisation during the pandemic compared to the pre-pandemic period. Since the Omicron variant, some general practices maintained a broad set of IPC measures, while others released most measures. HCWs’ future expectations on post-pandemic IPC implementation varied: some anticipated reduced implementation due to the desire to return to the pre-pandemic standard, while others expected IPC to be structurally scaled up during seasonal respiratory epidemics. Main contextual challenges included patient cooperation, staff shortages (due to infection), shortages of IPC materials/equipment, and frequently changing and ambiguous guidelines. Key lessons learned were enhanced preparedness (e.g., personal protective equipment supply), and a new perspective on care organisation (e.g., digital care). Main recommendations reported by HCWs were to strengthen regional collaboration within primary care, and between primary care, public health, and secondary care.

**Conclusion:**

HCWs’ experiences, perspectives and recommendations provide insights to enhance preparedness for future epidemics and pandemics, and sustain IPC in general practices. For IPC improvement strategies, adopting an integrated system-based approach that encompasses actions across multiple levels and engages multiple stakeholders is recommended.

**Supplementary Information:**

The online version contains supplementary material available at 10.1186/s12875-024-02451-z.

## Background

The coronavirus disease 2019 (COVID-19) has been a worldwide public health emergency that has put great pressure on healthcare workers (HCWs) and healthcare systems [1, 2]. General practitioners (GPs) were at the frontline of providing COVID-19-related healthcare while continuing to deliver routine care [3]. In addition, they played a key role in controlling the pandemic by administering vaccination programmes, using their information infrastructure to identify at-risk groups, and detecting new cases of COVID-19 [4].

Adequate infection prevention and control (IPC) within general practices is paramount for ensuring the quality of medical care and the safety of HCWs, patients, and the wider community [5, 6]. The outbreak of COVID-19 has necessitated significant changes in IPC protocols and procedures within general practices, including the increased adoption of personal protective equipment (PPE), the implementation of stringent triage protocols, the reorganisation of patient flow, the screening of patients, and altered practice layouts [7, 8]. Previous studies across different countries have also indicated increased adoption of digital care including telephone and video consultations in primary care settings [9–12].

Due to the pandemic, fewer face-to-face consultations took place and non-COVID-19 care was downscaled, leading to reduced access to primary care and delayed non-COVID-19 care [13]. Staff shortages caused by infections further strained capacity and access to care. Additionally, patients’ avoidance of primary care due to fear of infection exacerbated delays in receiving timely medical attention [14]. In addition, GPs experienced high workload and work pressure during the pandemic, due to the overwhelming COVID-19 care and administrative burdens [3, 15–17]. Moreover, medical assistants experienced high feelings of psychological burden during the first wave of the pandemic [18]. Next to these contextual challenges related to the pandemic, previous studies have identified practice and system factors to hinder IPC during the pandemic, such as inadequate GP practice building layout, limited IPC resources and materials, infrastructure constraints, and rapidly changing guidelines [19–21].

Despite the importance of IPC in general practices, IPC implementation in primary care settings remains an understudied topic [15, 19]. Previous studies mainly focused on the impact of the pandemic on routine care delivery in general practices, while the impact on IPC and the implications for future IPC implementation (post-pandemic) remains unaddressed. Therefore, this study aimed to examine HCWs’ experiences and perspectives regarding the implementation and organisation of IPC in general practices before and during the COVID-19 pandemic, and address its implications for post-pandemic IPC implementation. Furthermore, as interventions that incorporate the input and experiences from HCWs are more likely to be successful [22], we aimed to identify recommendations reported by HCWs to improve and sustain IPC.

The findings of this study can inform future IPC improvement strategies which can contribute to general practices’ preparedness for future seasonal respiratory epidemics or pandemics and help sustain IPC in primary care settings.

## Methods

### Setting: COVID-19 pandemic and organisation of Dutch general practices

Throughout the COVID-19 pandemic, the implementation and organisation of IPC changed, driven by fluctuations in infection pressure and disease burden. In periods prior to the Omicron variant, there was high infection pressure and disease burden, while the overall burden of disease has decreased since the introduction of the Omicron variant as a result of higher immunity due to prior infections and vaccinations [23]. Therefore, to assess the implementation and organisation of IPC, we made a distinction in the interviews between IPC implementation in pre-Omicron variant periods (2020–2021) and periods during the Omicron variant (2022-interview).

In the Netherlands, the organisation of general practices includes a network of GPs organised regionally. These regional GP networks serve as umbrella organisations to provide regional primary care policy and are responsible for organising out-of-hours care. Their main purpose is to promote collaboration, share best practices, and coordinate efforts to ensure the delivery of effective and efficient healthcare services.

### Study design

A qualitative study was conducted involving semi-structured, in-depth interviews. These interviews were performed during two data collection periods: pre-COVID-19 pandemic and during the pandemic (mainly Delta and Omicron variant periods). In-depth interviews aim to gain a detailed understanding of the research objectives from the study participants’ perspectives and personal experiences [24]. Data reporting was guided by the COnsolidated criteria for REporting Qualitative research (COREQ) guidelines [25] [See Additional file 1].

### Participant selection

Participants consisted of GPs and medical assistants from general practices located mainly in the southern region of the Netherlands, specifically in Limburg. Given the diversity in IPC procedures and responsibilities of GPs and medical assistants, our study aimed to select both groups of HCWs to obtain a comprehensive understanding of various professional experiences, perspectives, and recommendations regarding IPC in general practices.

To select participants, convenience sampling with snowball methods were adopted [26, 27]. A study invitation was communicated in a newsletter of the regional antimicrobial resistance care network [28]. Furthermore, we requested enrolled participants to recruit additional participants among their colleagues. A conscious effort was made to introduce diversity in our sample across participant characteristics, including sex, age groups, and years of work experience. Moreover, we sought heterogeneity in practice characteristics, including practice size (both large and small), practice type (private practices and health centres with multiple GPs), and practices located in both rural and urban areas. After HCWs were willing to take part in our study, interviews were scheduled, and study details were shared, after which an informed consent form was signed. In case there was no initial response to the invitations, a maximum of three reminders were sent via email or telephone. Participant selection persisted until data saturation was attained [29].

### Data collection

Data collection occurred during two study periods: (1) prior to the COVID-19 pandemic (hereafter named pre-pandemic), from July 2019 to February 2020; (2) during the COVID-19 pandemic (hereafter named during the pandemic), from July 2022 to February 2023 (during periods of Delta and Omicron variants). Semi-structured, in-depth interviews were conducted, primarily held in person at the professional’s respective general practice. Interviews were audio-recorded. In response to the pandemic, and the increase in online meetings, we also allowed online interviews, as per HCWs’ preferences. The interviews pre-pandemic were administered by MH (PhD student), and the interviews during the pandemic were led by FH (PhD student), with an additional researcher (RH) present to co-facilitate and observe. This co-observer facilitated the collection of supplementary non-verbal data, such as body language, by making field notes. Participants were unacquainted with the interviewers in both data collection periods. At the beginning of each interview, interviewers introduced themselves as public health researchers and clearly stated the aim of the study, emphasising the importance of capturing a wide range of experiences, including negative reflections on IPC in general practices (during the COVID-19 pandemic). This was intended to foster an environment where participants felt comfortable sharing real-life experiences and authentic opinions without feeling the need to provide socially desirable responses.The interviews pre- and during the pandemic were guided by a topic list with open-ended questions [See Additional file 2]. Open questions were designed to encourage participants to express their feelings and thoughts freely about each question [30]. The topic list for the interviews pre-pandemic included two main topics: (i) implementation of IPC, and (ii) recommendations to improve IPC. As we were interested in the influence of the pandemic on IPC and its implications for future IPC implementation (post-pandemic), the following main topics were included in the topic list for the interviews during the pandemic, in line with research objectives: (i) implementation and organisation of IPC, including changes during the pandemic and future expectations (post-pandemic), (ii) contextual challenges, (iii) lessons learned, and (iv) recommendations to improve and sustain IPC. We also gathered data on participant characteristics such as occupation, age, and years of work experience, as well as general practice characteristics like size, type, and location. A multidisciplinary team — including GPs, infection control professionals, and researchers — contributed to the development of the topic lists. During the first interviews, the topic lists were pilot tested, and remained mostly unchanged, with only the question sequence being adjusted.

We employed an iterative approach for data collection, meaning data was collected concurrently with participant selection and analysis to determine data saturation [31].

### Data analysis

Audio-recorded interviews were transcribed verbatim by a professional transcription service company. Transcripts were systematically coded using ATLAS.ti 9 software for qualitative analysis. Data were analysed by thematic analysis, using both inductive and deductive approaches, following the six-step method by Braun and Clarke [32]: (1) familiarisation of data (2), generation of codes (3), combining codes into themes (4), reviewing themes (5), determine significance of themes, and (6) reporting of findings. The deductive analysis was guided by our predefined topic lists and employed a multilevel system framework. This approach enabled us to examine themes and concepts across multiple system levels: micro (patient, HCW), meso (organisation, GP practice), and macro (contextual factors, e.g., healthcare system-related factors). During analysis, we adopted a synthesis-oriented and overarching approach to compare the qualitative findings from the two data collection periods. This entailed a rigorous examination of the interconnectedness between codes and themes across both study periods. The process of coding was performed iteratively, persisting until no further codes emerged. To deepen our understanding and interpretation of the data, we compared field notes with transcripts. These field notes played a role in capturing contextual information and identifying substantial verbal expressions, nonverbal cues, and emotional undertones within interview passages throughout the coding process [33]. Consequently, our analysis encompassed both manifest and implicit facets of the qualitative data, yielding a more detailed and all-encompassing analysis. The process of coding was conducted independently by two researchers (FH and MvH for the pre-pandemic interviews, and FH and HV for the interviews during the pandemic), and discrepancies were discussed until a consensus was achieved.

## Results

For the interviews pre-pandemic, 14 of the 19 invited HCWs (74%) participated, and for the interviews during the pandemic, 22 of the 24 invited HCWs (92%) participated (Table 1). Main reasons for non-participation were time constraints. Two individuals participated in both study periods. There was some overlap with participants from the same GP practices: for the interviews pre-pandemic, three practices had two participants each. For the interviews during the pandemic, two practices had two participants each. During the pandemic, 9 interviews were conducted online and 13 interviews in person. On average, the interviews pre-pandemic had a duration of 38 min (range 24–53 min), equal to the interviews during the pandemic (range 20–53 min). Data saturation occurred after 12 interviews pre-pandemic and 20 interviews during the pandemic.

**Table 1:** Participant characteristics of the interviews conducted pre-pandemic (*n* = 14) and during the pandemic (*n* = 22)

Participant characteristics	*n* (%) / M (min-max)	
	Interviews pre-pandemic (*n* = 14)	Interviews during the pandemic (*n* = 22)
*Occupation*		
General practitioner	10 (71.4%)	13 (59.1%)
Medical assistant	4 (28.6%)	9 (40.9%)
*Sex*		
Female	7 (50%)	15 (68.2%)
Male	7 (50%)	7 (31.8%)
*Working experience (years)*	21 (1–32)	12 (6–30)
*Age (years)*	50 (22–65)	39 (25–64)

*Abbreviations.* M = mean, Min = minimum, Max = maximum

The themes include an overarching interpretation of the data including pre-, during and post-pandemic reflections, with the main focus on the latest interview findings during the pandemic. Six main themes were identified in qualitative analysis: (1) shift towards comprehensive IPC implementation and organisation during the pandemic compared to the pre-pandemic period (2), diversity in IPC implementation and organisation since the emergence of the Omicron variant (3), post-pandemic diversity in future expectations on IPC implementation and organisation (4), contextual challenges (5), lessons learned, and (6) recommendations reported by HCWs to improve and sustain IPC. The findings were underpinned by quotations, selected based on their contextual depth and richness.

### (1) Shift towards comprehensive IPC implementation and organisation during the pandemic compared to the pre-pandemic period

**Fig. 1:**
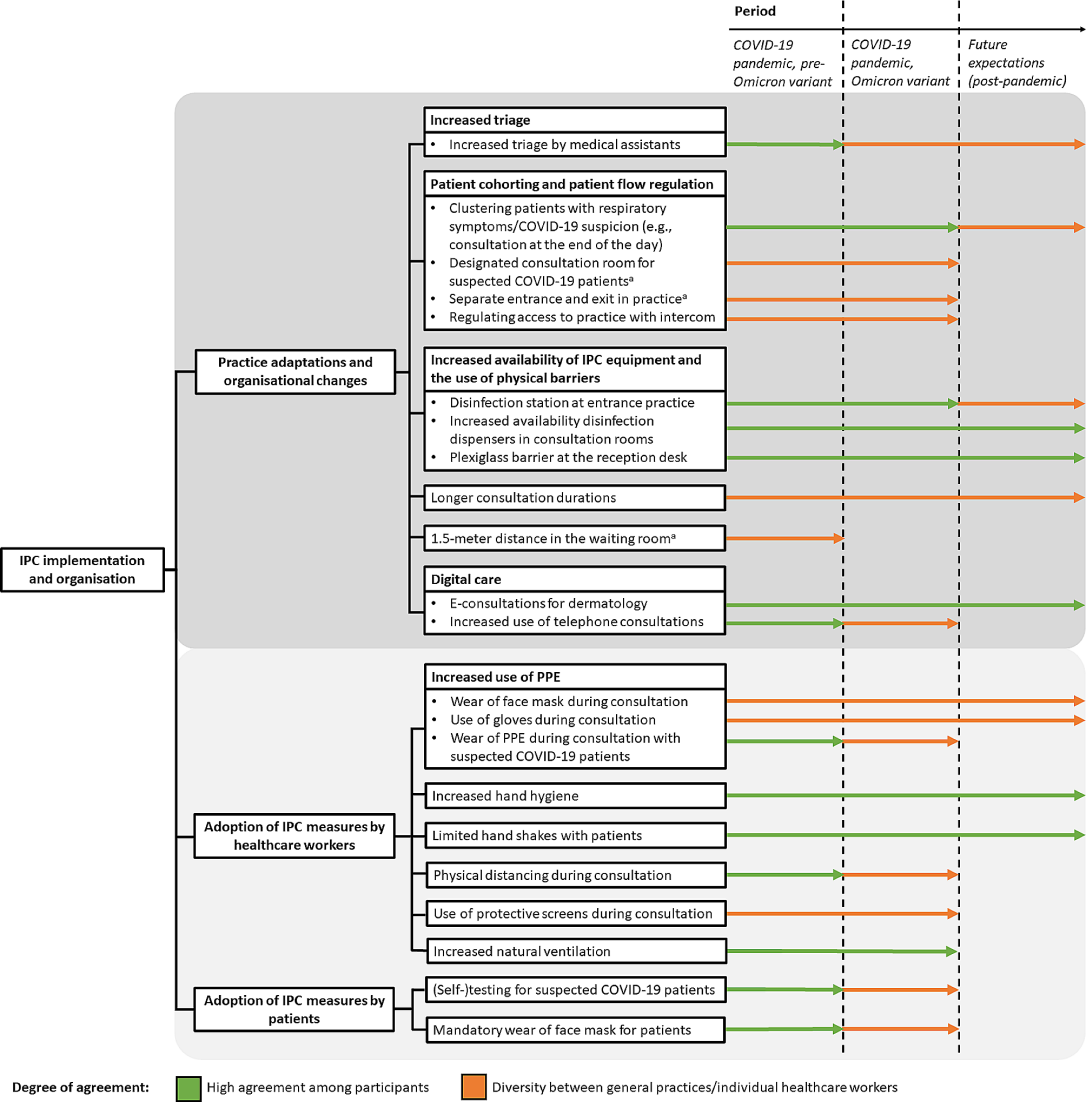
Overview of IPC implementation and organisation per period (pre-Omicron variant, during Omicron variant, and future expectations [post-pandemic]) and the degree of agreement among participants (high agreement among participants [green] vs. diversity between general practices/individual healthcare workers [orange]) ^a^ These practice adaptations were not possible due to building limitations. Yet, healthcare workers sought appropriate solutions when faced with building-related obstacles (e.g., letting suspected COVID-19 patients wait in their cars instead of placing them in the waiting room or centralise care for suspected COVID-19 patients at an out-of-hours GP service or a single GP practice) *Abbreviations*. IPC = infection prevention and control, PPE = personal protective equipment

Pre-pandemic, IPC was mainly associated with small surgical procedures, antimicrobial resistance, and prescription of antibiotics. During the pandemic, comprehensive IPC measures were implemented. Pre-Omicron period (2020–2021), all general practices followed COVID-19 guidelines, and implemented a similar set of recommended IPC measures (Fig. 1), including enforced hand hygiene practices, no handshakes with patients, physical distancing, increased natural ventilation, disinfection stations at the entrance, 1.5-meter distance in the waiting room, increased triage, patient cohorting (e.g., separate consultation for suspected COVID-19 patients, often scheduled at the end of the day, conducted in a designated COVID-19 consultation room), patient flow regulation (e.g., separate entrance and exit), plexiglass barrier at the reception desk, increased use of digital care (especially telephone consultations), increased availability of disinfection dispensers in consultation rooms, mandatory wear of face masks for patients, and (self-)testing for suspected COVID-19 patients. In addition, some general practices regulated access to the practice with an intercom, and few practices were completely closed (due to infections among staff). GPs sometimes centralised care for suspected COVID-19 patients at an out-of-hours GP service or a single GP practice, due to fear of contamination among GPs, PPE shortages, staff shortages, or an increasing number of patients with a COVID-19 infection.

*“During the pandemic, access to the facility was regulated. An intercom was installed for people to announce themselves at the door, whether they had an appointment or not. Suspected COVID-19 patients were led to a designated consultation room, which was distinct from the waiting area, disinfected regularly, and all non-essential items such as paintings were removed due to concerns about aerosol transmission. Precautions included opening windows to ventilate with clean air for at least 15 minutes. Suspected COVID-19 patients needed to wear a face mask, and physicians wore disposable protective clothing, a face mask and gloves when seeing these patients.”* (P22, woman, medical assistant, 40-45y, during the pandemic), *“Video calling fails to gain traction, with minimal interest from our patient population. Telephone calls are the preferred mode of communication, as this is familiar to patients, avoiding the complexities of links and codes.”* (P10, man, GP, 45-50y, during the pandemic), “*Hand sanitisers are now placed throughout the building, which we did not have before. It is now on every desk, so everyone is sanitising their hands more frequently and paying attention to it.”* (P17, woman, medical assistant, 25-30y, during the pandemic).

Specific IPC measures could vary slightly among different general practices while the general principles of IPC implementation and organisation were the same during the pandemic (pre-Omicron period). A few participants mentioned that a designated room for consultations with suspected COVID-19 patients or maintaining 1.5-meter distance in the waiting room was not possible due to building limitations. Yet, HCWs mentioned seeking appropriate solutions when faced with building-related obstacles. For instance, they would use a different building for suspected COVID-19 patients, schedule patients at the end of the day, or to let patients wait in their car.

There were small differences between individual GPs’ use of PPE and protective screens. Some GPs reported using protective screens on desks, while other GPs indicated not using them because they had close patient contact during physical examinations or had the possibility of maintaining distance through the width of the desk. During the first waves of the pandemic, GPs reported to see patients in full disposable protective clothing. Nevertheless, a few GPs mentioned wearing white coats instead of disposable gowns, often for sustainability considerations.

### (2) Diversity in IPC implementation and organisation since the emergence of the Omicron variant

Since the Omicron variant, some practices maintained a comprehensive set of IPC measures, while others released most measures. The reason to maintain a comprehensive set of IPC measures was to prevent other infectious diseases besides COVID-19: *“Since the Omicron variant, no changes were made regarding IPC. It is still needed when seeing patients with flu or respiratory symptoms, not only COVID-19-related. We have to be cautious with high-risk groups. Supplies like disinfectant and alcohol, white coats (even for assistants) and desk-mounted protective screens are still used.”* (P21, woman, medical assistant, 40-45y, during the pandemic). Most HCWs mentioned still having the disinfection station at the entrance of the practice during the Omicron variant: *“The disinfection station at the entrance remains for now, at least until spring, after the infections have passed – influenza, adenoviruses, etc. We might consider reinstating it next fall, during the respiratory season.”* (P13, woman, GP, 35-40y, interviews during the pandemic). *“Patients still make frequent use of the disinfection station at the entrance.”* (P21, woman, medical assistant, 40-45y, during the pandemic). Furthermore, HCWs stated that there was still an abundance of disinfection dispensers in the consultation rooms and within the general practice, significantly more than pre-pandemic. The majority of participants mentioned that increased triage was still being performed and patients with respiratory symptoms were still being asked to perform self-tests. However, a few HCWs mentioned no longer applying additional triage. Multiple participants mentioned still clustering suspected COVID-19 patients: *“When patients present respiratory or suspected infectious symptoms, they are required to take a test and call if it is positive before the appointment. Masks are mandatory at the practice for patients. We (as HCWs) might wear masks based on their symptoms. Positive patients are seen at the end of the day. If they have severe symptoms, they are asked to wait outside until called in.”* (P15, man, GP, 45-50y, during the pandemic). Some HCWs mentioned still having protective screens in the consultation rooms. All participants reported retaining the plexiglass barriers at the reception desk: *“We still have the plexiglass barrier at the reception desk because, during conversations, people tend to talk with a lot of saliva, so it is beneficial to have the glass barrier.”* (P20, woman, medical assistant, 25-30y, during the pandemic). According to national guidelines, the 1.5-meter distance in the waiting room was released in all practices from the Omicron variant onwards. However, a few HCWs mentioned trying to maintain a 1.5-meter distance in the consultation room as much as possible. HCWs, especially medical assistants, also reported continuing to clean, sanitise, disinfect, and ventilate (particularly the designated COVID-19 consultation room) more frequently during the Omicron variant than in the pre-pandemic period. Regarding digitalisation, several participants mentioned still using digital care, primarily through telephone consultations rather than video calls. However, some mentioned discontinuing telephone consultations since the Omicron variant and reported seeing all patients in person. The majority of HCWs reported still using e-consultations for dermatological conditions or check-ups. Other organisational changes that some HCWs still adopted were regulating the practice with an intercom and adjusting the scheduling of appointments (allowing more time between consultations and distributing appointments among GPs to reduce crowding in the practice). A few HCWs reported structurally implementing longer consultation durations in the practice: “*Initially, patients had many questions and anxieties about small matters, requiring the physician’s time. Therefore, we decided to keep the fifteen-minute consultation [instead of 10 minutes] to assist patients effectively, allow them to express themselves clearly, provide reassurance, address concerns, and find solutions.*” (P21, woman, medical assistant, 40-45y, during the pandemic).

Regarding the adoption of IPC measures, several HCWs mentioned making the choice to provide additional protection for themselves and wearing a face mask in certain situations: *“I always wear a face mask during consultations with patients having respiratory symptoms. Also, with children, infants, RS virus, and similar cases. I will continue doing this for now.”* (P13, woman, GP, 35-40y, during the pandemic). Whereas others mentioned not wearing a face mask or only wearing one when seeing a patient who tested positive. A few participants reported that HCWs within the practice continued to wear face masks consistently during the Omicron variant when delivering patient care: *“In the consultation room, we wear FFP2 masks. Physicians also have a protective screen. If they need to listen to the lungs, they wear an FFP2 mask for added safety. Assistants also continue to wear masks, especially during blood testing procedures.”* (P22, woman, medical assistant, 40-45y, during the pandemic). Multiple HCWs also mentioned wearing gloves more frequently during physical contact with patients compared to the pre-pandemic period: *“Currently, I hardly perform any physical examinations without gloves. Like dentists, it should be routine to always wear gloves.”* (P9, man, GP, 50-55y, during the pandemic). On the other hand, a few HCWs mentioned not wearing gloves more frequently during patient contact, yet applying additional hand hygiene: *“I only use gloves when absolutely essential, such as for tending to wounds, not for respiratory issues. However, I sanitise my hands a lot more often than before.”* (P16, woman, medical assistant, 25-30y, during the pandemic). The majority of participants mentioned no longer shaking hands with patients: *“I used to shake hands with all forty patients I saw in a day, upon arrival and departure. It has been three years without it, and I doubt it will ever return. It feels odd to shake hands now.”* (P9, man, GP, 50-55y, during the pandemic). However, the interviews revealed that HCWs still engaged in occasional handshakes, based on the initiation of patients: “*I no longer shake hands … Except when patients actively offer a hand, especially elderly, then I do not decline, but I do not initiate them myself*.” (P15, man, GP, 45-50y, during the pandemic). Even during the Omicron variant, all HCWs reported increased hand hygiene practices compared to pre-pandemic periods, particularly in terms of increased use of hand sanitiser: “*I am conscious of hand hygiene. I use Sterillium [brand of hand sanitiser] after each contact*.” (P1, woman, GP, 50-55y, during the pandemic).

Certain IPC minimum requirements applied to all HCWs within general practices (e.g., mandatory face mask for suspected COVID-19 patients), but further implementation was professional-dependent and based on the decision-making process of the GP: *“The implementation of IPC depends on personal choice, except for high-risk patients and patients with confirmed COVID-19 infection. A colleague who is part of the high-risk population uses a FFP2 mask and gloves during consultations with patients with respiratory symptoms. Colleagues that are less concerned see patients without PPE.”* (P3, man, GP, 40-45y, during the pandemic).

### (3) Post-pandemic diversity in future expectations on IPC implementation and organisation

HCWs frequently mentioned being relieved when certain measures can be relaxed and they can return to the pre-pandemic standard: “*COVID-19 will soon become like the flu, which is why we should treat it like the flu and not overreact.”* (P1, woman, GP, 50-55y, during the pandemic). However, other participants perceived it as important to apply IPC measures to other infectious diseases beyond COVID-19, and expected to scale up IPC during seasonal respiratory epidemics: “*It will be of added value to start wearing a face mask during a flu season as well. Gloves, more frequent handwashing, and sanitising too. We already have hand sanitisers everywhere, but it might be good to have patients to continue using them. It is important to maintain these practices, also in light of preventing future outbreaks of other infectious diseases, even influenza.”* (P14, man, GP, 50-55y, during the pandemic). Despite variation in future expectations, all HCWs agreed there should be a structural focus on facilitating IPC in terms of logistical matters (e.g., PPE supply) and the physical environment (building and layout) of both existing and new GP practices. In addition, participants uniformly expressed to maintain increased hand hygiene practices (mainly disinfection), limit handshakes with patients, and maintain the plexiglass barrier at the reception desk. Moreover, several participants expressed the expectation of increased glove use during physical examinations, and the placement of hand sanitising stations at the entrance during the flu season. Additionally, during flu seasons, some participants anticipated increased triage by medical assistants, the implementation of separate consultations for patients with respiratory symptoms, and the wear of face masks during consultation with patients exhibiting respiratory symptoms or during close physical contact.

An overview of the implementation and organisation of IPC is illustrated in Fig. 1, including during and post-pandemic reflections, and participants’ agreement.

### (4) Contextual challenges

HCWs encountered various contextual challenges regarding IPC implementation and organisation during the pandemic (Table 2). Challenges related to the pandemic were in the following areas: patient, (human) resources, regulatory framework and communication, workload, team factors, and access to care. Patient resistance to measures, lack of understanding, and a lack of cooperation were frequently mentioned: *“Convincing people to comply with IPC poses a significant challenge, with patient resistance being the most important barrier.”* (P7, man, GP, 30-35y, during the pandemic). Some participants also mentioned that certain patients intentionally withheld information about symptoms or test results, or provided misleading information. Nonetheless, HCWs reported that the patient group exhibiting resistance was relatively small, and they also received many positive responses and gratitude from patients. Shortages of IPC resources and materials (e.g., PPE and tests) and staff shortages (due to infection) were also often reported: “*Staff shortage is an issue, leading to more phone calls, and less strict and precise triage or patient education. Resource and equipment scarcity was a major concern during the first waves of the pandemic.”* (P7, man, GP, 30-35y, during the pandemic). The frequently changing and sometimes unclear and ambiguous guidelines were also reported to pose a significant challenge. Additionally, guidelines were often offered (too) late and some practices implemented additional IPC measures on their own initiative. In addition, discrepancies in communication between the National Institute for Public Health and the Public Health Service led to confusion and variations in policies between practices: *“Across general practices in the region, there were often differences in IPC implementation, leading to friction. Patients would hear that other practices were implementing different measures, which felt odd at times.”* (P17, woman, medical assistant, 25-30y, during the pandemic). Furthermore, the blending of professional roles with personal lives sometimes led to dilemmas: *“It is quite challenging and confusing when there are personal and professional opinions, and no clear professional guidelines to follow. Especially regarding social contact outside of work, for instance, during Christmas, it is hard to navigate personal choices that may affect work and lead to ethical dilemmas.”* (P6, woman, medical assistant, 35-40y, during the pandemic). Personal choices regarding vaccination caused polarisation in the team. Furthermore, many HCWs identified increased workload, primarily driven by increased administrative tasks. Another challenge was reduced accessibility with delayed routine care and missed healthcare needs: *“I believe the elderly, a vulnerable group, have struggled, since they often seek care for minor issues due to feelings of loneliness or social isolation.”* (P1, woman, GP, 50-55y, during the pandemic), *“We even have patients who have become fearful of healthcare, avoiding seeing a GP due to concerns about getting sick at the practice. This has led to delays in seeking care for serious infections or underlying conditions.”* (P15, man, GP, 45-50y, during the pandemic). HCWs also hinted at challenges related to the primary care sector or healthcare system, facing difficulties even before the pandemic, like labour market shortage, high workload and time constraints during consultations: “*Time constraints during consultations and high workload does not make IPC easy. We have ten minutes, and everything needs to be done within that time.”* (P1, woman, GP, 30-35y, pre-pandemic).

### (5) Lessons learned from the pandemic

A main lesson learned is the new perspective on practice organisation (Table 2). The realisation that digital care is achievable was often mentioned: *“We have learned that telephone consultations and e-consultations are effective, which is new for us. We have also learned that we must adapt, be flexible, and respond quickly, making adjustments regarding consultation hours and planning.”* (P16, woman, medical assistant, 25-30y, during the pandemic). Additionally, HCWs emphasised the importance of regulating patient flow and access to the practice, and the need to triage more effectively and extend the duration of consultations from 10 to 15 min were mentioned as lessons regarding practice organisation. Another mentioned lesson was increased preparedness, mainly related to the supply of PPE: *“We have learned to be better prepared, proactively ensuring an adequate supply of PPE, such as protective clothing and ensuring timely restocking of face masks.”* (P3, man, GP, 40-45y, during the pandemic). Moreover, some HCWs reported that protocols were developed during the pandemic, which could be consulted in the event of future outbreaks or epidemics. However, other HCWs reported that the required IPC measures are not documented in written protocols but are “fresh in their minds”. A few HCWs emphasised the importance of implementing IPC measures earlier than previously done to avoid practice closures as an important lesson learned.

Most interviewed HCWs were particularly positive about their ability to maintain continuity of care during the pandemic. They also reported feeling positive about how they collectively dealt with the situation, with a prevailing sense of togetherness during the pandemic.

### (6) Recommendations reported by healthcare workers to improve and sustain IPC

A key recommendation (Table 2) mentioned by the majority of HCWs is the importance of strengthening existing regional collaboration in primary care for logistical matters such as resource allocation and coordination of patient flow, preferably facilitated at a higher, central level: *“Patient flow should occur and be facilitated at a higher level. Patient groups with similar symptoms or medical conditions should be treated at a central location, such as dedicated COVID-19 facilities such as COVID-19 hotels or out-of-hours GP services.”* (P3, man, GP, 40-45y, during the pandemic), *“We had to figure out where to order our supplies ourselves. This could be more centralised, for example, managed by the care group.”* (P15, man, GP, 45-50y, during the pandemic). HCWs stated that having such a central treatment location also reduces pressure on regular consultations, allowing for better continuity of routine care. Another important recommendation was to strengthen regional interdisciplinary collaboration between primary care, public health, and secondary care (i.e., between general practices and public health services, laboratories, and hospitals) for information provision on (regional) infectious disease trends: *“Structural information provision by the Public Health Service and regional laboratories on regional infection incidence and prevalence trends regarding infectious diseases would be helpful, as it creates a sense of urgency when infection rates increase. Not just for COVID-19, but also for other diseases.*” (P7, man, GP, 30-35y, during the pandemic). Some HCWs expressed the need for the development and communication of practical, clear, and uniform IPC guidelines: *“Clear guidelines and policies are needed. Just, 5 ground rules to follow. A decision tree would be helpful. Lengthy documents like the current Dutch LCI guidelines are unclear and confusing, especially when rules change frequently.”* (P6, woman, medical assistant, 35-40y, during the pandemic). In addition, some participants highlighted the importance of considering practical feasibility and maintaining a balance with other healthcare services when implementing IPC measures in general practices: “*Practical feasibility at the workplace is key. Patient care, especially acute care, cannot be compromised. Our profession is much broader than just IPC. If all focus has to be on that (IPC), it comes at the expense of other aspects of care provision.”* (P2, woman, GP, 35-40y, during the pandemic). In line, several HCWs emphasised the importance of evidence-based measures that align with the risks in primary care: “*For meaningful progress, it is crucial to measure and understand the infection risks within general practices, as well as the effectiveness of IPC measures. IPC should be evidence-based.”* (P10, man, GP, 45-50y, during the pandemic). Additionally, some HCWs expressed the need for increased collaboration and dialogue between the government and healthcare institutions for the upscaling and downscaling of IPC measures. A few participants mentioned the importance of continuously assessing the needs of HCWs to maintain sustainability and feasibility of IPC implementation.

**Table 2:** Overview of contextual challenges, lessons learned from the pandemic, and recommendations to improve and sustain IPC in general practices, reported by general practitioners and medical assistants (*n* = 14, interviews pre-pandemic; and *n* = 22, interviews during the pandemic)

**Contextual challenges** *Contextual challenges related to the pandemic* • Patient-related factors: patient resistance to measures, lack of understanding and cooperation among patients, patients withholding or providing misleading information about symptoms or test results • Materials and resources: shortage of IPC materials/equipment (during the first wave(s) of the pandemic) • Human resources: staff shortages (due to infection) • Regulatory framework and communication: frequently changing, unclear and ambiguous guidelines, discrepancies in communication between governmental public health agencies • Team factors: blending of professional roles with personal lives, polarisation in team • Workload: increased workload due to the administrative burden of the pandemic • Access to care: reduced accessibility of primary care and missed healthcare needs *Contextual challenges related to the primary care sector and healthcare system* • Setting: time constraints in consultation and high workload • Human resources: labour market shortage in primary care	
**Lessons learned from the pandemic** • New perspective on care organisation, including integration of digital care, regulation of patient flow, improved triage processes, extended duration of consultations (from 10 to 15 min) • Increased preparedness through adequate supply of personal protective equipment and development of IPC protocols during the pandemic • Importance of implementing IPC measures earlier than previously done to avoid practice closures	
**Recommendations to improve and sustain IPC** • Strengthen existing regional intrasectoral collaboration in primary care for logistical matters (for resource allocation and coordination of patient flow) • Strengthen (regional) intersectoral collaboration between primary care, public health, and secondary care (i.e., between general practices and public health services, laboratories, and hospitals) for information provision on (regional) infectious disease trends • Development and communication of practical, clear, and uniform guidelines • Ensure that IPC remains feasible and balanced with other aspects of healthcare delivery	

*Abbreviations*. IPC = infection prevention and control

## Discussion

In this qualitative study, we evaluated HCWs’ experiences and perspectives regarding IPC in general practices before and during the pandemic, and its implications for post-pandemic IPC implementation.

We identified a more comprehensive implementation of IPC since the pandemic than pre-pandemic, which is consistent with findings of previous studies [8, 19, 34, 35]. Still, since the emergence of the Omicron variant, IPC implementation varied between general practitioners. This is in line with previous qualitative findings that have demonstrated the significant impact of individual preferences and decision-making processes of GPs on IPC implementation [8, 20]. This study demonstrated post-pandemic diversity in the future expectations regarding IPC implementation. Yet, our findings suggested that HCWs recognised the benefits of maintaining some IPC practices post-pandemic such as increased hand hygiene practices. This partially parallels findings in Australian primary care settings, which indicated that HCWs were inclined to maintain increased hand hygiene, the use of digital care and the wear of face masks by staff and patients [8].

The identified contextual challenges in this study are similar to prior studies; challenges include staff shortages as a result of infections and subsequent sick leave [13], the availability of PPE including medical masks [14], and the frequently changing IPC requirements and regulations during the pandemic [8, 21, 36]. The development and implementation of supportive policies, surveillance and reporting system activation (e.g., dashboards), availability of PPE, staff training, and workforce augmentation have been recommended to enhance pandemic preparedness of primary care [18, 37]. In addition, previous qualitative findings have demonstrated an experienced lack of clarity and ambiguity in the guidelines during the pandemic [36]. It is important to minimise redundancy of information across different governmental agencies, enhance the clarity of communication, and ensure the provision of consistent and clear guidance in future pandemics [21, 38]. Furthermore, our findings hinted at the challenges and potential adverse effects of limited access to care and missed care, which is consistent with concerns about the continuity of care during the pandemic [14, 39]. Moreover, our study’s findings demonstrated challenges with patient cooperation in light of COVID-19 control measures. At the same time, our findings and other studies also indicated expressions of gratitude, understanding, and appreciation of patients towards GPs during the COVID-19 pandemic [16].

One of the main lessons learned identified in our study is the new perspective on care organisation. An example is digital care or telemedicine (remote consultation) [9–12], which presents opportunities for aspects like chronic disease management and monitoring [36]. It also raises concerns, such as the absence of physical examinations and in-person interactions, potentially impacting physician-patient relationships [10]. Dutch GPs seem unlikely to continue the extensive use of remote consultations mainly because of workflow, time and cost considerations [40, 41]. Notably, the GPs in our study highlighted that regional intrasectoral (between general practices) and interdisciplinary collaboration (between primary care, public health and secondary care) is possible and needed, and should be maintained and strengthened. The pandemic accelerated awareness of the importance of interprofessional collaboration, and communication and coordination between primary care and public health authorities [11, 16, 21, 36]. Therefore, IPC needs to be addressed at multiple system levels, to reflect the complexity of healthcare environments. This requires the involvement of multiple stakeholders and organisations, emphasising the need for system commitment and interdisciplinary collaboration [42]. Recognising the interplay between policy, culture, systemic support, and individual behaviour is important to optimise IPC [19, 20]. The establishment of robust (infra)structural frameworks serves as a catalyst for driving behavioural changes at the individual professional level.

### Strengths and limitations

The strength of this study is that it incorporates the perspectives and experiences of HCWs, thereby providing valuable insights into daily work practices and its context. Furthermore, this study compares HCWs’ perspectives before and during the pandemic, thereby contributing to further insights into the topic and evolution of IPC in general practices.

This study is also subjected to several limitations. Firstly, convenience sampling methods were used to select participants, which could potentially lead to the introduction of selection bias [43]. One should note that HCWs in other parts of the Netherlands and other countries may have had different experiences during the pandemic. However, the inclusion of a diverse range of HCWs across various demographics and general practices aimed to mitigate this bias. Secondly, it proved challenging to recruit GPs and medical assistants for interviews, primarily because their heavy workloads, which were exacerbated by the pandemic. Factors such as staff shortages and staff turnovers further challenged recruitment efforts. Consequently, differences in participant characteristics emerged between the interviews conducted pre-pandemic and during the pandemic. These differences can be explained by the healthcare workforce demographics. During the pandemic, a higher proportion of our participants were medical assistants, who tend to be younger and have fewer years of working experience than GPs. Although there were no notable differences between the two professional groups regarding their experiences and perspectives regarding IPC, the variations in participant characteristics between the two data collection periods should be taken into account when interpreting our findings. Thirdly, in terms of recall bias, retrospective reflections on the COVID-19 period were conducted during phases of a relatively lower burden of disease compared to the initial waves of the pandemic. Despite this concern, the recency and impact of the pandemic likely enhanced memory recall, minimising this bias.

### Implications for practice

The COVID-19 pandemic prompted a re-evaluation of IPC organisation in primary care. This urgency has placed IPC at the forefront of healthcare agendas during the pandemic. Moving forward, it is crucial to embed IPC within general practices, harnessing the lessons learned during the pandemic. Given the ongoing risk of infection transmission post-pandemic, particularly during seasonal respiratory epidemics, systematic implementation of a comprehensive set of IPC measures remains relevant. Moreover, these IPC actions can also be considered for gastrointestinal epidemics, as they pose similar challenges in terms of IPC.

The findings of this study highlighted the need for enhanced regional collaboration within primary care (intrasectoral collaboration) and between primary care, public health, and secondary care (intersectoral collaboration). This calls for a stronger integration of IPC within the broader healthcare system. The establishment of robust (infra)structural systemic frameworks is important herein. Based on the findings of our study, we recommend that these frameworks should initially focus on (1) fostering intersectoral collaboration regarding information provision on infectious disease trends, and (2) intrasectoral collaboration within primary care regarding logistical matters.

Continuous communication and information sharing between primary care, public health, and secondary care is essential to enhance IPC. It is recommended for Public Health Services and regional labs to disseminate and share information on infectious disease trends in the region (transdisciplinary knowledge sharing), particularly during seasonal respiratory epidemics. The implementation of robust information exchange mechanisms, such as a platform or newsletter, enables real-time sharing of epidemiological data, emerging threats, and best practices. This collaboration enhances the ability to detect, monitor, and promptly respond to infectious disease outbreaks, fostering a proactive and unified response to emerging infectious diseases. Furthermore, clear, standardised, and uniform guidelines or regulatory frameworks should be communicated through established processes, involving regular updates disseminated through platforms, (interdisciplinary) meetings, or newsletters. Enhanced collaboration among different disciplines and organisations in the form of regular interdisciplinary meetings or a dedicated platform for asking questions may contribute to less ambiguity [44].

The optimisation of logistical aspects, such as resource allocation and coordination of patient flow, within primary care is critical for enhancing overall infectious disease preparedness and response capabilities. It is noteworthy that many of these collaborative structures proved effective during the pandemic. As we transition beyond the pandemic, it is imperative to underscore the importance of preserving and fostering these collaborative efforts among general practices in the same region. The exchange of personnel remains an adequate strategy during epidemics or pandemics to ensure sufficient staffing levels. Furthermore, the supply of IPC materials and equipment could benefit from a higher level of coordination, possibly at the regional level. To further optimise resource allocation and patient management, the establishment of centralised treatment facilities (e.g., COVID-19 hotel or out-of-hours GP service) is recommended during outbreaks with peak infection rates. Existing (region-based) GP networks are important in facilitating and sustaining these efforts. By fostering continued collaboration and providing a structured framework for communication and resource sharing, these networks may play a key role in maintaining a resilient primary care system.

Our study’s findings regarding the diversity in IPC implementation and organisation since the emergence of the Omicron variant further highlight the importance of harmonising IPC at a collective level (i.e., regional level). It is recommended that HCWs and other institutions establish harmonised IPC protocols and guidelines that can adapt to various infectious disease scenarios. An initial recommendation is to develop a practical toolbox for IPC implementation during seasonal respiratory epidemics. This toolbox should include decision-making tools for HCWs, offering clear guidance on which IPC measures to implement in which situations [19]. It should also provide specific ‘signals’ or indicators on when to scale up measures. The development of this IPC toolbox should be a collaborative effort involving active engagement with HCWs from general practices. This collaboration presumably enhances the effectiveness and compatibility of these tools with current work practices and routines at the workplace [20].

To successfully optimise and sustain IPC in general practices, it is important to acknowledge that the primary care sector is currently encountering significant challenges, primarily as a result of a shortage of personnel and high workload [45]. A previous study has highlighted the importance of supporting the workforce, communication, and the development of integrated care to overcome these challenges [44].

## Conclusions

Insights from HCWs’ experiences, perspectives and recommendations provide valuable *lessons learned*, which will contribute to enhanced *preparedness* for future epidemics or pandemics and sustain IPC in general practices. Main recommendations reported by HCWs are to strengthen regional intrasectoral collaboration within primary care and intersectoral collaboration between primary care, public health, and secondary care. To optimise and sustain IPC in general practices, it is advised to adopt an integrated system-based approach by acting on multiple levels and engaging multiple stakeholders.

### Electronic supplementary material

Below is the link to the electronic supplementary material.

Supplementary Material 1

Supplementary Material 2

## Data Availability

The datasets used and analysed during the current study are available from the head of the data-archiving of the Public Health Service South Limburg on reasonable request. Interested researchers should contact the head of the data-archiving of the Public Health Service South Limburg (Tamara Kleine: tamara.kleine@ggdzl.nl) when they would like to re-use data.
