# Supplementary material for: Infection prevention and control in Dutch general practices before and during the COVID-19 pandemic and its implications for pandemic preparedness and seasonal respiratory epidemics: a qualitative study on lessons learned
Source: BMC Prim Care. 2024 Jun 20;25:222. doi: 10.1186/s12875-024-02451-z (PMC11191277; doi:10.1186/s12875-024-02451-z)
Supplement: Supplementary file 2 — Supplementary Material 2 [file 12875_2024_2451_MOESM2_ESM.pdf]

Additional file 2: Interview topic guides for the interviews pre- and during the COVID-19 pandemic

Topic guide for the interviews pre-pandemic

**Background variables**	1. Could you introduce yourself?<br>o Age, gender, occupation, years of (work) experience.
	2. Could you tell me about your general practice (or the practice where you work)?<br>o Do you work solo or in a group practice?<br>o Are there specific patient groups you have more contact with than others?
	3. What comes to mind when you think about infection prevention and control (IPC)?<br>o What role did IPC play during your education/studies?<br>o Does your practice offer IPC education/are you taking IPC courses or training?<br>o Are you aware/familiar with IPC guidelines (e.g., hygiene guidelines) in your general practice?<br>i. To what extent do these guidelines play a role in your work? (hand hygiene, personal protective equipment, clothing regulations, hygienic working environment, resources and materials)<br>ii. Are these guidelines adequate? (e.g., comprehensibility, practicality, procedural clarity)<br>o How do you acquire IPC information?
**IPC implementation**	4. To what extent does IPC play a role in your work?<br>o What aspects of IPC do you apply? (hand hygiene, personal protective equipment, clothing regulations, hygienic working environment, medical waste disposal, antibiotic prescription behaviour)<br>o Could you share some experiences with IPC (measures)?

	5. To what extent does IPC play a role at an organisational level? (e.g., policy, formal agreements)<br>o How much attention is given to IPC?<br>o Do you think enough priority is given to IPC?<br>o Who deals with/are involved in IPC in your organisation?
**Recommendations**	6. Do you think there are changes needed with regards to IPC in your organisation? And if so, what would you recommend?
	7. What needs to change so you can focus more on IPC?
**Additional comments**	8. Do you have any (additional) questions or comments for us? Or additional experiences or opinions to share?
Abbreviation: IPC infection prevention and control	

<u>Topic guide for the interviews during the pandemic</u>

**Introduction**	1. Could you introduce yourself?<br>o Age, gender, occupation, educational background, years of (work) experience.
	2. Could you tell me about your general practice (or the practice where you work)?<br>o Do you work solo or in a group practice?<br>o Are there specific patient groups you have more contact with than others?
**Changes in IPC implementation due to the COVID-19 pandemic**	3. To what extent has the COVID-19 pandemic influenced domains/aspects of IPC? (hand hygiene, clothing requirements, personal hygiene, use of personal protective equipment, waste disposal, environmental cleaning, IPC resources/materials)<br>4. To what extent has the COVID-19 pandemic influenced physical and organisational changes regarding IPC? (telephone triage, digital/telephone consultations, separate COVID-19 consultation, patient flow regulation)<br>5. How is IPC integrated in the practice? (protocols, organisational priority, IPC training, communication of guidelines)

	o To what extent has anything changed as a result of the COVID-19 pandemic?
**Challenges**	6. Do you experience challenges in implementing IPC? If so, what?<br>7. Have the challenges you have experienced changed due to the COVID-19 pandemic? (patient-related, at organisational level)
**Future expectations**	8. What is the impact of the decrease in COVID-19 diagnostics on the various domains/aspects of IPC?<br>9. How will the implementation of IPC be handled in the future? Which measures will be retained, in which situations? (Scenarios: seasonal COVID-19, flu and cold season, absence of COVID-19, emerging COVID-19/new pathogenic variant)
**Lessons learned**	10. Looking back on the past period, what are you proud of regarding IPC? What went/goes well?<br>11. Looking to the future, what are the lessons you have learned and will apply to your future actions regarding IPC?<br>12. What needs to be considered more or given more attention in the field of IPC?<br>13. Who should play a role/be involved in this facilitation/change?<br>14. What are things you would have done differently with the knowledge you have now? Would you have made different considerations?<br>15. What does this mean for the future? Will you take these considerations into account in the future?
**Recommendations**	16. What would you recommend to make/keep IPC feasible in general practices?
**Additional comments**	17. Are there any other things/experiences you would like to share regarding IPC?<br>18. Are there any other points we have not discussed that you would still like to address?<br>19. Do you have any further questions or comments for us?
Abbreviation: IPC infection prevention and control	
